# Ethical Aspects of Personalized Research and Management of Systemic Inflammatory Response Syndrome (SIRS) in Children

**DOI:** 10.3390/ijerph20010470

**Published:** 2022-12-28

**Authors:** Elisa Groff, Marcin Orzechowski, Catharina Schuetz, Florian Steger

**Affiliations:** 1 Institute of the History, Philosophy and Ethics of Medicine, Ulm University, 89073 Ulm, Germany; 2 Paediatric Immunology, Medical Faculty “Carl Gustav Carus”, Technic University Dresden, 01307 Dresden, Germany

**Keywords:** systemic inflammatory response syndrome, SIRS, child, clinical ethics, research ethics, personalized medicine

## Abstract

Systemic inflammatory response syndrome (SIRS) is a life-threatening condition with nonspecific symptoms. Because of that, defining a targeted therapy against SIRS in children and adults remains a challenge. The identification of diagnostic patterns from individualized immuneprofiling can lead to development of a personalized therapy. The aim of this study was to identify and analyze ethical issues associated with personalized research and therapy for SIRS in pediatric populations. We conducted an ethical analysis based on a principled approach according to Beauchamp and Childress’ four bioethical principles. Relevant information for the research objectives was extracted from a systematic literature review conducted in the scientific databases PubMed, Embase and Web of Science. We searched for pertinent themes dealing with at least one of the four bioethical principles: “autonomy”, “non-maleficence”, “beneficence” and “justice”. 48 publications that met the research objectives were included in the thorough analysis, structured and discussed in a narrative synthesis. From the analysis of the results, it has emerged that traditional paradigms of patient’s autonomy and physician paternalism need to be reexamined in pediatric research. Standard information procedures and models of informed consent should be reconsidered as they do not accommodate the complexities of pediatric omics research.

## 1. Introduction

Systemic inflammatory response syndrome (SIRS) is a defense response of the organism to an insult such as infection, systemic inflammation or trauma [1]. It is caused by life-threatening organ dysfunctions due to a dysregulated immune response and can lead to multi-organ failure. The symptoms of SIRS are non-specific: it is precisely this lack of knowledge of the underlying cause that makes the choice of the right therapy correspondingly difficult [2]. In the majority of cases, management of the condition is based on clinical features and routine laboratory parameters. However, the limitation of this approach lead to a dramatic reduction in the percentage of recovery [3,4].

Children and adolescents can also become severely ill with SIRS due to a sudden and uncontrolled overreaction of their own immune system. This is a life-threatening condition that can affect any child. Indeed, the mortality rate for children admitted to the intensive care unit may substantially increase due to the onset of severe SIRS criteria [5,6]. For example, sepsis is one of the leading causes of pediatric death worldwide [7]. In 2005 adjusted criteria for SIRS in children were established for the first time [8]. However, despite adaptation of criteria for pediatric populations, heterogeneity of symptoms and reliance on conventional examination methods pose a challenge for proper diagnosis and therapy in pediatric SIRS patients.

A possible solution for lack of suitable therapy for SIRS is early stratification and personalized treatment of patients based on their biomarkers. The variation of immune responses in SIRS can be explained by genomic variants [9,10]. Therefore, generating personalized immunoprofiles of the patients and identifying diagnostic patterns can enable development of a tailored immunotherapy for patients with SIRS in the context of personalized medicine. This can improve the quality of medical care for pediatric and adult patients in the long term.

Personalized medicine—or precision medicine (PM)—takes into account variations in individual genes, environmental contexts and lifestyles of individual patients [11]. Analysis of these factors, based on large-scale biologic databases and often conducted with the use of digital algorithms—such as machine learning or artificial intelligence (AI)—allows for more accurate and efficient diagnosis and treatment of specific diseases [12]. This is at odds with a one-size-fits-all approach that applies the same treatment options to all patient groups indiscriminately. Developing a tailored treatment to SIRS, instead, holds out the promise of an improvement in the clinical management of SIRS patients, such as the reduction of side effects and socio-economic burden that SIRS brings with it.

An ethical analysis of issues related to research and personalized therapy of pediatric patients with SIRS has not yet been pursued. Therefore, the objective of this study was to (i) identify ethical issues that arise when conducting research on children and adolescents with SIRS, and (ii) propose recommendations to implement these ethical considerations in personalized research on pediatric SIRS.

## 2. Materials and Methods

This work is an analysis of ethical issues raised by immuneprofiling and guiding patient tailored treatment of systemic inflammatory response syndrome (SIRS) in children. The investigation applies the principle-oriented approach of Beauchamp and Childress, which is the cornerstone methodology for biomedical ethics in health practice [13].

For the purpose of our analysis, we began with a systematic literature search to identify the key themes for evaluating ethical implications in the clinical research and therapy of SIRS in pediatric populations. The search was carried out in the databases of scientific literature PubMed, Embase and Web of Science. The goal of the search was to identify publications that address ethical challenges and issues in the research and therapy of SIRS in children. Due to the dearth of literature assessing ethical aspects in the treatment of SIRS in children, no time frame was given in the first place. The research protocol followed the recommendations for conduct and reporting of systematic literature reviews [14].

**Figure 1:**
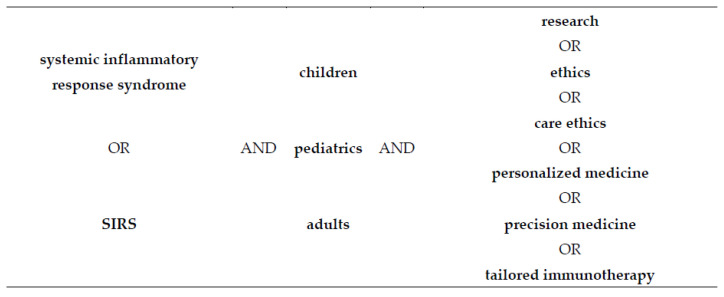
Combination of keywords applied to the systematic literature search.

The search algorithm combined the following keywords: “systemic inflammatory response syndrome” or “SIRS” with the keywords “children” or “pediatrics” or “adults” and “research” or “ethics” or “care ethics” or “personalized medicine” or “precision medicine” or “tailored immunotherapy”. The search used different combinations of keywords and accounted for variations in terminology. For example e, we searched with both the keyword “SIRS” and the exact phrase “systemic inflammatory response syndrome”. Likewise, we searched with both keywords “children” and “pediatrics” as well as with “personalized” and “precision medicine” (Figure 1).

**Figure 2:**
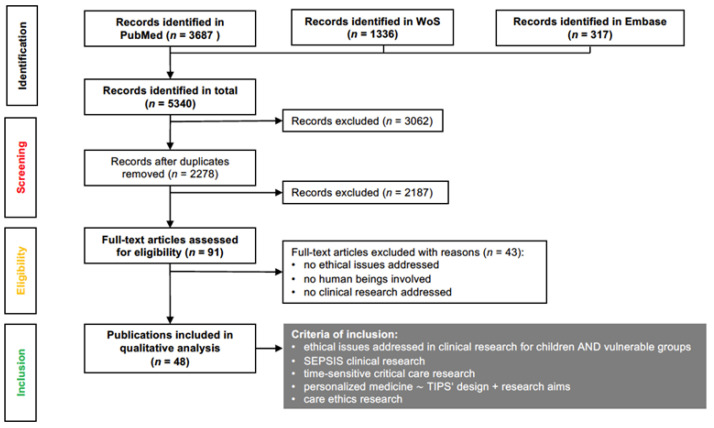
Flow chart detailing literature search and criteria of inclusion.

We conducted a multi-field search in Embase, and an advanced search in PubMed and Web of Science. The search algorithm yielded 5340 results. After removing 3032 duplicates, we proceeded to double screening the titles and abstracts of 2278 publications. A complete dual review of full-text search results was then performed. Excluded were 2187 publications which do not address the inclusion criteria. Ultimately, 91 publications from 1998 to February 2022 were classified as relevant and read in full. In the end, 48 publications that met the research objectives were included in the thorough analysis (Figure 2).

Due to the novel nature of aimed-specific research on SIRS, publications dealing with ethical aspects of other clinical research on children and other vulnerable groups were also included in the ethical analysis; for example, case reports and precision medicine studies whose research aim and design are similar to those of TIPS. In particular, as sepsis has been long defined as the presence of SIRS in addition to a suspected or documented infection, publications that address ethical aspects of clinical trials of sepsis were also included. Indeed, the morbidity, diagnostic and research goals of sepsis with the recent integration of an omics approach are similar to those of SIRS [15]. In addition, studies addressing time-critical care research, pediatric acute clinical trials and emergency research were taken into consideration, as well as publications concerning care ethics which is central to clinical pediatric research [16].

Publications which meet the research objectives were included in the thorough analysis. Relevant information for the research objectives was extracted in the research papers by applying the method of thematic analysis. We searched for pertinent themes that deal with at least one of the four bioethical principles according to Beauchamp and Childress, that is autonomy, non-maleficence, beneficence, and justice. The main aim of the ethical analysis was to find out what principles are affected in clinical research on personalized therapy of SIRS in children and adolescents, so that ethical questions arise. Thus, the first step was to screen publications for selection of units of information, which we defined as ethical issues. We defined ethical issues as a violation of an ethical principle or a conflict between at least two ethical principles. The second step was then to categorize the ethical issues according to ethical reference categories, namely the four biomedical principles. By applying such a principled framework, we related the spectrum of ethical issues that emerged from the literature review to the four moral principles in order to determine whether they were consistent or in conflict with these bioethical categories. The principles did not have to be explicitly stated. The results were then summarized, structured and discussed in a narrative synthesis. To reduce biases and omissions, articles were double examined for inclusion.

## 3. Results

48 publications that met the research objectives were included in the thorough analysis. In this section we report the ethical issues as identified in the literature. Table 1 below shows the applied principled framework for ethical issues according to Beauchamp and Childress’ four bioethical principles of “autonomy”, “non-maleficence”, “beneficence” and “justice”.

### 3.1. Autonomy

With regard to the principle of autonomy, the ethical issue of informed consent has been variously addressed in the literature [8,16,17,18,19,20,21,22,23,24]. It has been pointed out that more attention should be paid to the experience of research participants, as patients and their families need more than mere professionalism within the process of informed consent. This leads to the conviction in the literature that (a) researchers have an obligation to provide continuous counseling and explanation throughout the study—when and if needed—and (b) participants have the right to withdraw from the study at any time without being required to provide their reasons. Another ethical issue deemed worthy of consideration in pediatric research is the way in which medical information is disclosed to patients, relatives or legal representatives [17,19]. The process of providing research participants with all available details, properly and effectively, is defined as a moral duty which (a) sets an ethical training for doctors involved in clinical studies as a requirement and (b) refines the definition of medical professionalism as comprising ethical competence [16,25] and trained communication skills. A communicative competence should teach doctors to tailor their language and medical information to patient’s individual level of health literacy, as well as to that of their families [16], and to respect patient’s preferences in the framework of confidentiality [17,23,26,27,28,29]. Ethically informed medical professionalism is also described in the literature as fundamental to shared decision-making [16,23], which ensures that patients are supported to reach a decision that suits them and the preferences of those involved. From the analysis of the selected publications following aspects have also emerged with the aim of respecting patient’s autonomy: researchers in clinical trials have the obligation (a) to protect the privacy of research participants’ personal data [1] and (b) to disseminate results to participants, in conferences and/or in publications [31,32,33,34]. Ultimately, the issues of deferred informed consent and waiver for ethics approval have arisen as an ethical challenge [18,26,35].

### 3.2. Beneficence

Trained communication skills have also been defined as the obligation of physicians to act for the benefit of the patient [25,27]. Indeed, the need for qualified agents to provide benefits—i.e., positive beneficence—and to balance benefits, risks and costs, i.e., utility, is the aim of continuing medical education [16,25,27,36,37]; whereas the goal of clinical research is to be twofold, namely reaching a scientific understanding leading to sound clinical interventions and designing a common ground in research for future collection and interpretation of data [15,19,28,35,41,42,44,45,46,47]. Another major ethical issue germane to ethical research with children, and which has been associated in the literature with the principle of beneficence, is medical paternalism. It has been argued that it is a fine line clinical pediatric research continuously walks between justified paternalism and a set of medical practices prone to abuse patients by overriding their choices [18,26,54].

### 3.3. Non-Maleficence

With regard to the principle of “non-maleficence”, one of the major ethical issues that has come to the foreground in the literature is the debate over alternatives models of informed consent for research with biological samples, e.g., broad, waived or deferred consent [7,18,24,25,27]. Furthermore, it has been pointed out that ethical standards for clinical research need to be clearly defined in multicentre intensive care trials so that all clinical researchers involved have the same competencies to fulfil the same obligations [25,27]. Germane to the obligations of non-maleficence which ought to be met in omics research, there is not only that of “not causing harm” to patients by negligence in the conduct of research, but also that of “not imposing risks” on patients. Consensus is also that many of the skills clinical researchers should be equipped with as a requirement for avoiding harm extend beyond clinical competence [16,24,26,27]. These include a discretionary effort to put empathy into action and thus connect to research purposes by mitigating patients’ fears [25], and ad-hoc communication skills for physicians who benefit enormously from training in how to navigate emotionally charged conversations [16,27]. Finally, a shared concern in the literature is the misleading use of language in form and contents in healthcare research. Language has been described as a powerful tool with the potential of addressing patients’ concerns and misconceptions about research, yet with the power to cause harm when it comes to approaching sensitive research-related issues with patients [16,19,24,26]. Indeed, inadequate medical information may lead to patient’s inadequate understanding which in turn, may result in an impasse in informed consent [16,17,18,19]. Hence, researchers should strive for a patient-tailored information for improved research participation, reduce withdrawal from research and minimal risk of harm [24,26,27].

### 3.4. Justice

The present analysis has highlighted that patterns of racial, ethnic, gender, religious and economic inequalities massively affect the principle of justice in clinical research [2,16,27,41,49]. Indeed, recent studies have shown that differences exist in SIRS in adults before trauma in relation to age and sex, but also race [49]. This raises the question whether the criteria of patient inclusion in a study should be adjusted according to age, sex, comorbidities and chances of survival [41,51]. Furthermore, it points to a better allocation of resources [16,33,36,38,41,46,48,49,50] that complies with human rights [20,52]. Justice in the context of pediatric ethics is called upon to find a balance among medical utility, progress of science and prospect of success [51] by avoiding a possible conflict of interest [16,17,18,20,26,44,53,55]. Finally, two thorny ethical challenges have arisen in respect of the principles of both beneficence and justice, namely (i) the selection criteria for enrolling patients in a trial and (ii) the ranking of patients for high-cost trials, such as genetic testing, according to their chances of survival. Here we are faced with a dual model for selecting patients according to prognosis or prediction and the associated costs: down one path are patients selected according to prognosis, which means that selection focuses on those patients who are more likely to die; down the other path are patients selected according to prediction, which means that selection focuses on those patients who are more likely to respond positively to treatment [41].

## 4. Discussion

From the analysis of the results extracted in the literature review it has emerged that traditional paradigms of patient’s autonomy and physician paternalism need to be reexamined in pediatric research. Furthermore, standard information procedures and models of informed consent need to be reconsidered as they do not accommodate the complexities of pediatric omics research [18]. A particular emphasis is also suggested to be placed on practical strategies aimed at handling biased criteria of inclusion in research. Finally, it has been declared necessary for pediatric studies to be subject to an independent ethical review in order to ensure the quality and impact of research data [40].

Our analysis has highlighted an array of ethical issues that ought to be considered when conducting personalized research and therapy of SIRS in pediatric population. With respect to the principle of autonomy, it is of utmost importance to mention that the research and treatment of pediatric patients involve a replacement of the classical relationship dipole—patient vs. physician—with a triangle of actors—child/adolescent vs. legal guardians vs. physician [56]. Accordingly, traditional paradigms of patient’s autonomy and physician paternalism need to be reexamined. Child/adolescent autonomy requires self-reliance on independent decision, free from parental or peer pressure. This is closely related to the questions whether children have the competence for reasoning and participating in decision-making that affect them in healthcare [57], under what circumstances this can be taken as an expression of a child’s autonomy, and whether this capability is gradually developed with age [58]. Legally, children’s consent is bound to statutory age limit stated by national jurisdictions. Generally accepted in European and North American clinical practice is that children are capable of assent, i.e., affirmative agreement taking place after an informed consent procedure in which a minor takes part. In this scenario, their legal representatives carry the responsibility for the consent, as a recent European Union wide study has shown [59]. However, an estimation of age limits for competency of minors shows that children of 11.2 years of age and above are potentially competent to consent to research participation [60]. A version of the MacArthur Competence Assessment Tool for Clinical Research (MacCAT-CR) modified for use with children has been adopted in this study for assessment of minors’ capacity to consent in clinical studies [60]. This instrument measures the four aspects of decision-making capacities: (i) understanding the disclosed information about the nature and procedures of the research; (ii) reasoning in the process of deciding about participation; (iii) appreciation of the effects of research participation on the patient’s own situation; (iv) expressing a choice about participation [59,61]. However, the caveat is that the age limit indicated in the study does not correspond to the age limit for consent by jurisdiction. Therefore, a structured assessment in form of MacCAT-CR can be only an instrument for clarification of a child’s developing autonomy. In order to empower and evaluate the capacity of a child to participate in decision-making that affects them, both healthcare professionals and legal guardians can start with listening to the child’s views. Similar examples regarding consent for vaccination are applied across Europe [62].

Closely connected to the question of a child’s developing autonomy is the ethical issue of medical paternalism. In research and therapy involving minors, paternalism is a set of attitudes and practices in which physicians, parents or both overrule a child’s wishes. Overprotective parenting can weaken the minor’s capacity for autonomous decisions [62]. Similarly, physician paternalism can limit parents’ and also children/adolescents’ decision-making through imposition following exclusion from the informed consent process [63].

Facilitating minor’s and parents’ autonomy in decision-making requires interaction through provision of information. This is particularly true for pediatric omics research. Challenges related to inadequate understanding of information during the informed consent process has been observed in both research [64,65] and therapy [66]. What is more, comprehension of research details involving minors is often reported as unsatisfactory [67]. Indeed, standard information procedures cannot accommodate the complexities of pediatric omics research. An additional level of complication stems from the involvement of children/adolescents and their cognitive abilities. An obligation to use age-appropriate terminology is a minimum requirement in pediatric research [68]. In order to achieve a better understanding, alternative methods of information with the use of enhanced paper forms, such as educational materials with simplified text, illustrations, narrative approaches, or multimedia, such as video presentations, apps, vignettes should be implemented [16,69,70]. Interactive tools, such as questionnaire with subsequent feedback for decision-makers, can be an excellent resource to assess the level of understanding of legal guardians and/or children [71,72]. When dealing with minors, employing questionnaires with interspersed assessment of comprehension can be of help [73,74]. Moreover, considering the principle of non-maleficence, doctors and researchers in clinical trials who have direct contact with patients should be trained not only in communication skills but also in how to navigate complex, emotionally charged conversations [16,27]. This is to avoid harm and additional emotional stress to the patient.

The inclusion of alternative models of informed consent, such as waived or deferred informed consent has been recommended to be implemented in pediatric acute care trials and time-sensitive critical care research when (a) there is not enough time to obtain informed consent, (b) if parents or legal representatives are not available, (c) if they are not aware of their child’s condition, or (d) they are too stressed to be approached [18,22]. In case of deferred informed consent, it has been suggested that when the patient regains capacity, the enrolment in the study be not reversed, but the patient or their legal representatives may subsequently refuse further participation, access their data, and be given the right to have their data deleted [26]. In order to reduce patient’s anxiety, respect patient wishes and ensure patient welfare, it has been recommended that (i) health professionals and researchers involved in clinical studies should be hired for their values not just for their skills, (ii) continuous counseling and mental health support be made available during the whole research process [26], and (iii) telephone assistance be put in train when physicians are off-duty. This could be made possible, for example, thanks to on-duty experienced nurses with access to patients’ health records who could provide immediate pertinent information and set-up the first available appointment with members of the research team [16].

With regard to the principle of beneficence, there are several considerations to be put forward when it comes to personalized research on pediatric SIRS. Methods of applied ethics can be implemented to inform pediatric clinical research and illuminate the ethical challenge of therapeutic misconception [19]. Indeed, therapeutic misconception may occur when research participants fail to understand that research is intended for group benefit in the first place and then for individual benefit; this means that research is conducted with the chief aim of creating scientific knowledge for society, and not of proving individualized medical treatment for research participants. This ethical challenge is tangential to the principles of informed consent and that of disclosure of risks and benefits. It raises the question of making language less ambiguous, using more appropriate information and excluding participants who are unlikely to derive direct benefit from participation in the research. Therefore, it has been deemed of help to invest in narrative medicine training to develop a deep form of active listening [16], as well as to train physicians and researchers involved in pediatric clinical trials in practical approaches to ethical issues [67] and in assessing and communicating risks and benefits of omics research from an ethical perspective [15,19]. To help the identification of ethical challenges in pediatric clinical trials with the aim of addressing those accordingly, it has been suggested that interviews and question surveys with different experts should be integrated within the research design. These have proved to be a valuable method when it comes to addressing practical ethical issues in research practice [16,67].

With regard to the issue of justice, our results have shown that the criteria of inclusion in the research with SIRS patients can be subject to bias. Violations of the principle of justice can be especially detected when digital algorithms and artificial intelligence are used in healthcare research. Underrepresentation or overrepresentation of specific patient groups within data set can lead to biases in form of class imbalance or unequal distribution of features [75]. For example, underrepresentation of certain population groups on the basis of gender or ethnic background has been observed in oncologic data sets. For this branch of research, class imbalance in omics data has been described not as the exception in the literature [76]. Moreover, imbalanced data sets lack the capture of social determinants of health, such as geographical or socioeconomic factors [77]. AI systems that rely on biased data of this kind thus lead inevitably to algorithmic bias. This means that the prejudice embedded in the AI decision will fake the predictive model in favor or against the person or the group that are overrepresented in the data set [75,78]. Therefore, bias in patient inclusion affect the downstream analysis of genomic and proteomic data sets which in turn, alter the credibility of the research outcome and the effectiveness of new treatment management strategies. Reducing inequalities and bias in clinical research should be achieved through seeking greater diversity among participants [16]. Furthermore, in order to optimize the allocation of resources in research, strategies should be implemented to invest in organizational culture and promote more empathy, e.g., availability of quiet spaces for families [16].

## 5. Conclusions

Multi-omics research holds a great promise to fill our understanding of pediatric SIRS. Accordingly, personalized therapy of pediatric SIRS can provide an effective solution to assess and improve children’s health. Yet, personalized research and therapy of SIRS in pediatric populations raise considerable ethical challenges. To date, there is no published study expressly addressing ethical issues related to personalized research on SIRS in children. The results of our analysis show that ethical considerations comprise all four bioethical aspects. Particular emphasis is placed on whether, and to what extent, minors have the capacity to make autonomous decision for themselves so as to participate in research aiming at personalized therapy. Moreover, the question of an adequate process of information that suits both minors and legal guardians involved in research is of particular significance. Therefore, based on the results of our analysis we propose the following recommendations for implementation in clinical practice: (i) individual case-to-case assessment of child/adolescent capacity for informed consent to research by means of structured competence assessment, e.g., with help of MacCAT-CR test modified for use with children; in case of estimated competency for consent, active involvement of minors in the decision-making process; (ii) use of alternative methods of information alongside standard information procedures, such as video presentations, apps, vignettes to improve understanding of provided information, especially for minors; (iii) assessment of level of understanding of provided information through interactive tools; (iv) training in ethical competence and communication skills for physicians involved in research and personalized therapy; (v) inclusion of heterogeneous patient groups in research in order to avoid gender, racial, religious and cultural disparities that may result in algorithmic bias.

## Figures and Tables

**Table 1:** Ethical issues identified in the literature.

Ethical Issue	Reference to the Literature	Bioethical Principles Affected
process of informed consent	[8,16,17,18,19,20,21,22,23,24]	Autonomy
disclosure of all available information to patients	[17,19]	Autonomy
Professionalism	[16,25]	Autonomy
trained communication skills	[16,24,26,27]	Autonomy Non-maleficence Beneficence
confidentiality and respect for patient’s wishes	[17,23,26,27,28,29]	Autonomy
shared decision-making	[16,23]	Autonomy
anonymization of personal data	[30]	Autonomy
dissemination of results	[31,32,33,34]	Autonomy
waiver for ethics approval	[18,26,35]	Autonomy
inadequate information	[7,25,26,30]	Non-maleficence
alternative informed consent for genetic analysis	[7,18,24,25,27]	Non-maleficence
continuing medical education	[7,36,37,38,39]	Beneficence
paternalism	[12,30,40]	Beneficence
goal of clinical research	[25,37,38,41,42,43,44,45,46,47,48]	Beneficence
Inequalities	[2,4,7,38,49]	Justice
allocation of resources	[7,36,39,41,44,46,48,49,50]	Justice
medical utility vs progress of science vs prospect of success	[51]	Justice
respecting human rights	[45,52]	Justice
legal issues	[3,7,12,13,30]	Justice
conflict of interests	[7,44,45,53]	Justice

## Data Availability

Not applicable.
